# Neural signatures of Trail Making Test performance: Evidence from lesion-mapping and neuroimaging studies

**DOI:** 10.1016/j.neuropsychologia.2018.03.031

**Published:** 2018-07-01

**Authors:** Andreja Varjacic, Dante Mantini, Nele Demeyere, Celine R. Gillebert

**Affiliations:** a Department of Experimental Psychology, University of Oxford, 15 Parks Road, OX1 3PH Oxford, United Kingdom; b Research Center for Movement Control and Neuroplasticity, University of Leuven, Tervuursevest 101 box 1501, 3001 Leuven, Belgium; c Department of Health Sciences and Technology, ETH Zurich, Winterthurerstrasse 190, 8057 Zurich, Switzerland; d Department of Brain and Cognition, University of Leuven, Tiensestraat 102 box 3711, 3000 Leuven, Belgium

**Keywords:** TMT, Set-switching, fMRI, VLSM, Mental flexibility

## Abstract

The Trail Making Test (TMT) is an extensively used neuropsychological instrument for the assessment of set-switching ability across a wide range of neurological conditions. However, the exact nature of the cognitive processes and associated brain regions contributing to the performance on the TMT remains unclear. In this review, we first introduce the TMT by discussing its administration and scoring approaches. We then examine converging evidence and divergent findings concerning the brain regions related to TMT performance, as identified by lesion-symptom mapping studies conducted in brain-injured patients and functional magnetic resonance imaging studies conducted in healthy participants. After addressing factors that may account for the heterogeneity in the brain regions reported by these studies, we identify future research endeavours that may permit disentangling the different processes contributing to TMT performance and relating them to specific brain circuits.

## Highlights

• The neural underpinnings of trail making test (TMT) performance remain unclear.
• We reviewed empirical evidence from healthy and brain-injured individuals.
• Regions in prefrontal and parietal cortex were found to mediate various TMT indices.
• There was a large variability in the brain regions reported across the studies.
• We suggest potential research avenues to unravel the neuroanatomy of the TMT.

## 1 Introduction

Originally conceived by the United States Army psychologists as a measure of general intelligence, the Trail Making Test (TMT) (e.g. Reitan and Wolfson, 1985) is a tool that is extensively used for the assessment of set-switching in brain-injured patients, defined as the ability to flexibly switch attention between competing task-set representations. The standardised version of the TMT comprises two task components, TMT-A and TMT-B. The TMT-A requires the participant to draw lines and connect circled numbers in a numerical sequence (i.e. 1–2–3 etc.). In the TMT-B, the participant is asked to draw lines to connect circled numbers and letters in an alternating numeric and alphabetic sequence (i.e., 1-A-2-B etc). The participant is instructed to complete both task components as fast and accurately as possible without lifting the pen from the worksheet. The typical administration of the TMT does not involve an explicit time constraint in the instructions, though a cut-off of 300 s is typically applied for the test discontinuation (Bowie and Harvey, 2006). The experimenter monitors task performance and in the event of error initiates error-correction.

TMT performance is typically indexed by the total time to completion (Bowie and Harvey, 2006). The TMT-A is thought to provide a baseline measure of psychomotor speed, visuospatial search and target-directed motor tracking and is administered first in sequence. The TMT-B is supposed to be matched to the TMT-A for low-level processes with an additional demand of set-switching. To isolate the latter component, corrected TMT-B scores are derived, obtained by calculating a difference (TMT-B – TMT-A), a ratio (TMT-B / TMT-A), or a proportional score [(TMT-B - TMT-A) / TMT-A] (e.g. Stuss et al., 2001; Muir et al., 2015). Empirical support for common low-level processes underlying TMT-A and TMT-B comes from studies reporting significant correlations between TMT-A and TMT-B outcome measures in both healthy (e.g. Arbuthnott and Frank, 2000) and brain-injured individuals (Kortte et al., 2002, Muir et al., 2015). However, the exact nature of the cognitive component that makes the TMT-B more “difficult”, as indexed by a completion time cost relative to the TMT-A, has been debated. There is evidence suggesting that the TMT-B induces heightened low-level visual search demands (Gaudino et al., 1995). For instance, the TMT-B involves longer distances between individual visual array elements compared to the TMT-A, which may place heightened demands on low-level visuospatial search and motor components (Gaudino et al., 1995). Alternatively, the performance on the TMT-B has been suggested to reflect higher-order processes based on a series of studies that validated the TMT-B outcome measures against widely used tests of executive functions, including tests of set-switching ability (Arbuthnott and Frank, 2000, Kortte et al., 2002). For instance, ratio, but not difference scores correlated with task-switch response times in a sample of young, healthy adults (Arbuthnott and Frank, 2000). Furthermore, raw TMT-B completion times correlated with the set-switching, but not the set-maintenance component of the Wisconsin Card Sorting Task (WCST) in a sample of neuropsychological patients (Kortte et al., 2002).

Whereas it does not directly contribute to the standard scoring protocol (Bowie and Harvey, 2006), performance accuracy may also be calculated and different types of errors can be defined. For instance, TMT-B *shifting* errors (failure to alternate between letters and numbers, e.g., connecting A-B…. or 1–2…) have been distinguished from TMT-B *sequencing* errors (failure to connect letters or numbers after a set shift in the correct order, e.g. connecting A-2-C… or 1-A-3…) (Klusman et al., 1989). Several studies have shown that error analysis may yield increased specificity in detecting cognitive impairment in clinical populations (e.g., Amieva et al., 1998; Stuss et al., 2001; Mahurin et al., 2006; Christidi et al., 2013; Kopp et al., 2015). However, other studies have questioned the clinical utility of TMT error analysis (Ruffolo et al., 2000, Klusman et al., 1989).

Multiple cognitive processes are likely to be engaged when performing the TMT (see Sánchez-Cubillo et al., 2009, for a review), implying that different brain regions may be important for successful completion of the task. This complicates the identification of the neural correlates of the TMT. In this review, we examine and synthesize evidence from lesion-symptom mapping studies conducted using the TMT in patients with brain damage and functional neuroimaging studies conducted in healthy individuals. Based on the reviewed findings, we suggest future research endeavours that may help a more precise identification of the neural circuits that underlie TMT performance.

## 2 Lesion-symptom mapping studies in patients with brain damage

Lesion-symptom mapping studies are conducted with the goal of identifying the brain regions that, when lesioned, are significantly associated with behavioural impairments (Bates et al., 2003, Rorden et al., 2007) (Table 1). Lesion-symptom mapping offers several advantages compared to traditional approaches such as lesion overlap and subtraction analysis, for relating structural lesions to cognitive deficits (see for instance, Vandenberghe and Gillebert, 2009). Most importantly, lesion-symptom mapping does not require the *a priori* categorization of patients into separate groups depending on the overall location of the brain damage. Instead, it enables one to examine whether individual brain regions or voxels can predict behavioural impairment, thereby increasing the anatomical precision of the analysis. This is particularly important when aiming at contrasting the results of lesion studies with functional activity maps obtained in healthy individuals. For this reason, the results of lesion overlap and subtraction studies examining the neural correlates of the TMT (e.g., Stuss et al., 2001; Yochim et al., 2007) were not examined here (see MacPherson et al., 2015, for a recent review of these studies).

**Table 1:** Characteristics and main findings of VLSM studies of the TMT. For each study, information about sample size, disease aetiology, lesion hemisphere, age, chronicity, TMT variant, VLSM outcome variable and identified clusters is provided.

**Study**	**Sample size**	**Aetiology**	**Lesion hemisphere**	**Age (M ± SD)**	**Chronicity (M** ± **SD)**	**TMT variant**	**Lesion-mapping outcome variable**	**Identified clusters**
Muir et al. (2015)	106; Chronic = 61, Acute = 45	Ischemic stroke	Not reported	Chronic = 64 ± 12 years Acute = 63 ± 14 years	Chronic = 423 ± 246 days Acute = 102 ± 19 days	Standard TMT	|TMT-B – TMT-A| difference score |(TMT-B – TMT-A)/TMT-A| proportion score |TMT-B/TMT-A| ratio score TMT-B error frequency	None.
Gläscher et al. (2012)	344; TMT subset = 236	Stroke (N = 253); Temporal lobectomy (N = 42); Focal surgical resection (N = 34); Encephalitis (N = 7); Focal pathology (N = 8).	Left (N = 174);	Not reported	Chronic	Standard TMT	VLSM of |TMT-B – TMT-A| difference score. VLSM conducted on standardised and covariate-corrected task residuals.	Left rostral anterior cingulate
			Right (N = 122);					
			Bilateral (N = 48).					
Barbey et al. (2012)	182	Penetrating head injury	Not reported	58 years	Chronic	TMT from Delis-Kaplan Executive Function System (D-KEFS)	VLSM of common variance shared across deployed executive tests	Regionally non-specific lesion effect including: Left lateral frontopolar cortex; Left anterior prefrontal cortex; Left dorsolateral prefrontal cortex; Left superior and inferior parietal cortex
Kopp et al. (2015)	30	Stroke (ischemic and haemorrhagic)	Right, frontal (N = 30)	60 ± 10 years	Acute (mean stroke to MR scan interval = 4 ± 3 days; mean stroke to CT scan interval = 3 ± 4 days).	Standard TMT	VLSM of TMT-A and TMT-B errors, VLSM of TMT-A and TMT-B raw completion time.	VLSM of TMT-B total errors: Right dorsolateral prefrontal cortex (37, 17, 32); Right frontal subgyral white matter (38, 2, 24); Right frontal subgyral white matter (34, 5, 32); No significant lesion effects detected for the completion time metric
Miskin et al. (2016)	27	Epilepsy (N = 8); Astrocytoma (N = 4); Cavernoma (N = 3); Oligodendroglioma (N = 3); Glioma (N = 2); Glial tumor (N = 1); Low-grade Glioma (N = 1); Ganglioglioma (N = 1); Tumor (N = 1); Meningioma (N = 1) Meningioma and Haemorrhagic Stroke (N = 1); Hamartoma (N = 1).	Left, frontal (N = 15); Right, frontal (N = 10); Bilateral, frontal (N = 2).	37 ± 11 years	Chronic (3 ± 3 years)	Standard TMT	VLSM of TMT-B raw completion time.	Left dorsomedial prefrontal
Varjacic et al. (2017)	144	Stroke (ischemic and haemorrhagic)	Left (N = 47); Right (N = 60); Bilateral (N = 37).	71 ± 15 years	Acute (5 ± 4 days)	TMT analogue from Oxford Cognitive Screen (OCS)	VLSM of accuracy score on the set-switching condition	Left insula

Lesion-symptom mapping can be carried out using a priori defined regions of interest (ROIs) or using a voxel-based approach. The ROI-based approach was used, for instance, in a multi-centre TMT study of acute and chronic stroke patients (Muir et al., 2015) to test whether brain areas previously associated with executive functions (i.e. dorsolateral prefrontal cortex, ventrolateral prefrontal cortex, dorsomedial prefrontal cortex, lateral parietal cortex and subcortical structures) were linked to poor performance on the TMT. Several corrected TMT-B completion time measures were used (including difference, ratio and proportion scores) as well as TMT-B *shifting* and *sequencing* errors. Notably, no significant association was found between lesions in the selected ROIs and these measures. However, a significant brain-behaviour relationship may exist in other brain regions or at a finer scale than the defined ROIs. To address these concerns, the voxel-wise lesion-symptom mapping (VLSM) approach can be employed, which uses the lesion status (lesioned/intact) at every given voxel to test for a significant difference in the distribution of the patients’ behavioural scores (Bates et al., 2003, Rorden et al., 2007). Several studies used VLSM to investigate the neural correlates of the TMT in patients with brain damage.

A large-scale VLSM study of the TMT provided evidence for the regionally specific involvement of the left rostral anterior cingulate in set-switching (Gläscher et al., 2012). Difference scores were used in the study, after eliminating contributions of verbal ability, visuo-spatial reasoning, verbal and visual memory. A further analysis quantifying the volumetric overlap between lesion effects from the VLSM analyses of other three tests of executive functions (including STROOP, WCST and Controlled Oral Word Association Test – COWA) showed that the left rostral anterior cingulate substrate was not exclusively related to the difference scores obtained with the TMT. In particular, lesions within the left rostral anterior cingulate predicted worse set-switching in the WCST, but not worse COWA-indexed verbal fluency and STROOP-indexed response inhibition. This finding extends previous TMT validation studies (Arbuthnott and Frank, 2000, Kortte et al., 2002) by providing indirect evidence in support of the argument that successful performance on the TMT-B requires set-switching.

Another large-sample VLSM study of the TMT examined war veterans with cortical lesions due to penetrating head wounds (Barbey et al., 2012). The common variance underlying performance was estimated across multiple tests of executive function, including the TMT variant of the Delis-Kaplan Executive Function System (D-KEFS) battery (Delis et al., 2001). An association was reported between left-lateralised, regionally non-specific prefrontal, insular, temporal and parietal lesion sites and standardized executive scores from the D-KEFS. A recent study of a chronic, frontal and aetiology-diverse brain-injured patient cohort suggested the prefrontal cortex to be lesioned when TMT-B performance was impaired (Miskin et al., 2016). More specifically, lesions within the dorsomedial prefrontal cortex were found to predict longer raw TMT-B completion times.

The above-mentioned VLSM studies evaluated chronic brain-injured patients. To the best of our knowledge, only two studies have used VLSM to evaluate TMT performance in acute stroke patients. Noteworthy, both studies used accuracy measures rather than completion times as dependent variable. In the first study, the acute stroke sample included 30 right-hemispheric patients with frontal lesion topography (Kopp et al., 2015). An association was found between circumscribed lesion sites within the right dorsolateral prefrontal cortex and TMT-B *sequencing* and *shifting* errors, respectively. In the second VLSM study, acute stroke patients without hemi-spatial neglect (Varjacic et al., 2017) performed the TMT analogue of the Oxford Cognitive Screen (OCS, Demeyere et al., 2015), which is an optimised test battery for the cognitive assessment of stroke patients. Specifically, participants were asked to connect large-to-small target shapes embedded in an array of non-target shape distractors (baseline condition, analogous to TMT-A) and to connect large-to-small target shapes in an alternating fashion (set-switching condition, analogous to TMT-B). The TMT variant of the OCS thus allows one to rule out number and letter sequencing deficits. The VLSM analysis was conducted on the total accuracy from the set-switching condition. The study showed that structural damage in the left insular cortex predicted lower accuracy scores in the set-switching condition above and beyond low-level visuospatial and motor demands of the baseline task.

## 3 Functional neuroimaging studies in healthy individuals

The TMT can be conceptualised as a visuo-motor sequence-tracking task that elicits an additional set-switching demand in the TMT-B condition. The motor response modality of the standard paper-and-pencil TMT implies that the speeded, coordinated and goal-directed motor behaviour is an important factor contributing to successful task performance (Schear and Sato, 1989). A challenge one faces in the adaptation of the TMT for the MR scanner involves adjustment of the motor response modality. Below we review the task-related (Table 2) and resting-state fMRI studies (Table 3) investigating the neural underpinnings of the TMT.

**Table 2:** Characteristics and main findings of fMRI activation studies of the TMT. For each study, information about sample size, age, TMT variant, fMRI task characteristics, behavioural effect, fMRI contrast and identified clusters is provided.

**Study**	**Sample size**	**Age (M ± SD)**	**TMT variant**	**fMRI task characteristics**	**Behavioural effect**	**fMRI contrast**	**Identified clusters**
Moll et al. (2002)	7	24 ± 9	Verbal TMT. Covert articulation of the numerical sequence (TMT-A) and number-letter alternating sequence (TMT-B) in response to the acoustically presented cue.	Block duration: 25 s Block repetition: 10 × TMT-A, 10 × TMT-B blocks Practice: administered in standard and verbal-TMT formats	Non-tractable due to covert task requirements.	v. TMT-B > v. TMT-A	L precentral gyrus (−44, −2, 38); L inferior frontal sulcus (−40, 23, 29); L middle frontal gyrus (−36, 38, 22); L dorsal premotor cortex (−31, −16, 50); L intraparietal sulcus (−35, −55, 34); L rostral supplementary motor area/cingulate sulcus (−6, 3, 49); R intraparietal sulcus (26, −55, 34)
Zakzanis et al. (2005)	12	29 ± 5	Motor TMT. Connect the trails in the scanner using the specially designed fibre-optic drawing device ("virtual stylus") and monitor performance simultaneously onscreen inside the scanner.	Block duration: 45 s Block repetition: 4 × TMT-A, 4 × TMT-B, 8 × motor baseline conditions. Practice: 5-min practice session on the virtual stylus	Significantly fewer trails connected in the TMT-B (M = 9 ± 1) compared to the TMT-A (M = 10 ± 1) (p < 0.01).	TMT-B > TMT-A	Left-lateralised cluster comprising middle frontal gyrus (−37, 9, 31); precentral gyrus (−34, 8, 37); cingulate gyrus (−13, 9, 28); superior frontal gyrus (−20, 23, 49); medial frontal gyrus (−15, 13, 46); insula (−37, −15, 10). Left-lateralised cluster comprising middle temporal gyrus (−61, −27, 7); superior temporal gyrus (−48, 41, 10). Right-lateralised cluster comprising cingulate gyrus (20, −20, 28); insula (27, −15, 22); paracentral lobule (15, −30, 43)
Jacobson et al. (2011)	16	23 ± 4	Computerised TMT (pc-TMT). Indicate the line orientation attached to each circled number/letter stimulus by a button press.	Block duration: 45 s Block repetition: 4 × TMT-A, 4 × TMT-B Practice: session administered in both written and computerised formats. Accuracy cut-off (90%) applied prior to entering the scanner.	No significant behavioural effect.	TMT-B > TMT-A	L middle temporal gyrus (−35, −68, 28); R precentral gyrus (31, −1, 31); R inferior middle frontal gyri (36, 34, −3)

**Table 3:** Characteristics and main findings of fMRI connectivity studies of the TMT. For each study, information about sample size, age, TMT variant, FC analysis and identified clusters is provided.

**Study**	**Sample size**	**Age (M ± SD)**	**TMT variant**	**FC analysis**	**Identified clusters**
Seeley et al. (2007)	21	35 ± 16	Standard TMT administered outside the scanner. |TMT-B – TMT-A| difference score used as a primary outcome measure.	Correlational analysis between |TMT-B - TMT-A| difference score and functional connectivity within the pre-defined executive network. Functional connectivity expressed as a z-score reflecting the relationship between the individual network seed time-series and overall network connectivity time-series.	L intra-parietal sulcus (−38, −78, 36); R intra-parietal sulcus (36, −80, 26).
Damoiseaux et al. (2008)	22	71 ± 6	Standard TMT administered outside the scanner. Raw TMT-B completion time scores used as a primary outcome measure.	Partial correlational analysis between intrinsic connectivity within the anterior default mode network and TMT-B raw completion time scores.	Anterior default mode network comprising clusters including superior frontal gyrus (x = −6, y = 66, z = 14); posterior cingulate (x = −8, 46, 34); bilateral angular gyrus (x = −44, y = −60, z = 30; x = 43, y = −62, z = 28).
James et al. (2016)	44 (TMT subset = 42)	31 ± 10	TMT from the Denis-Kaplan Executive System Battery (D-KEFS). Primary outcome measure: Age- and education-corrected difference completion time scores. Difference computed between number-letter switching condition and numerical sequencing TMT D-KEFS conditions (analogous to |TMT-B - TMT-A|)	Pairwise correlational analysis between 200 atlas-defined regions of interest	R ventrolateral prefrontal (39, 47, 6); L superior-parietal lobule (−15, −68, 53)

### 3.1 Task-related functional magnetic resonance imaging (fMRI) studies of the TMT

The first attempt to implement the TMT in an MR scanner involved the elimination of visual and motor components of the original TMT task (Moll et al., 2002). Participants were required to covertly articulate the number sequence (verbal analogue to the TMT-A condition i.e. “verbal TMT-A”) or the alternating number-letter sequence (verbal analogue to the TMT-B condition i.e. “verbal TMT-B”) in response to the acoustic cue (“count” versus “alternate”, respectively). Given the covert response modality, the behavioural scores were derived by the self-report probe after completion of the experiment. Specifically, participants were asked to recall their best performance scores for TMT-A and TMT-B conditions. Increased activation in the TMT-B relative to the TMT-A condition was observed in the left middle frontal gyrus, left precentral gyrus and bilateral intra-parietal sulcus.

Another fMRI study capitalised upon the motor requirements of the TMT by developing an MR-compatible fibre-optic tracking device, which allowed execution of complex motor behaviour and simultaneous onscreen performance monitoring inside the scanner (Zakzanis et al., 2005). A baseline condition was deployed to elicit non-target motor behaviour by asking participants to link up a sequence of empty circles. At the behavioural level, significantly fewer trails were connected in the TMT-B relative to the TMT-A condition. The neural analysis showed the TMT-B condition elicited greater activation relative to the TMT-A in a left-lateralised cluster comprising middle and superior frontal subdivisions, precentral gyrus and insular cortex. Whereas the behavioural pattern is suggestive of a lower response frequency in the TMT-B condition, neither the TMT-A nor the motor baseline condition was optimised to match the TMT-B condition in this respect. This raises the possibility that the reported neural findings in this study also reflect non-specific motor activation.

More recently, an fMRI study adapted the TMT for the scanner by developing a computerised TMT variant (i.e. “pc-TMT”) that retained the visual mode of presentation, but attenuated the motor component of the original test (Jacobson et al., 2011). Specifically, participants tracked a number sequence (“pc-TMT-A”) or an alternating number-letter sequence (“pc-TMT-B”) by indicating the orientation of a square attached to each circle with a button-press. The methodological strength of the computerised adaptation is that it allowed a precise collection of response times for each ‘connection’ in the sequence, permitting the quantification of the pc-TMT-B average “switch time”. Further, the estimation of the pc-TMT-B “switch cost” relative to the pc-TMT-A condition (behavioural study, Experiment 1; Jacobson et al., 2011) was used to inform the optimisation of the TMT-A baseline condition for the fMRI experiment (Experiment 2; Jacobson et al., 2011). By extension, the pc-TMT-A baseline condition involved a delay period (reflecting the pc-TMT-B minus the pc-TMT-A “switch cost”) added to each response in the sequence. The baseline optimisation was intended to equalise the response frequency and match the motor response profile across conditions. In contrast to the left-lateralised activation effects identified in the fMRI study of the “verbal” TMT (Moll et al., 2002), the pc-TMT-B versus pc-TMT-A comparison isolated right-lateralised activation in inferior frontal and precentral gyri.

### 3.2 Resting-state functional connectivity studies of the TMT

Resting-state functional connectivity (rs-FC) studies of the TMT capitalise upon the inter-individual variability of the behavioural scores by investigating how behavioural data recorded outside the scanner maps onto FC patterns in a task-free (i.e. resting-state) context. A first study investigating rs-FC in relation to TMT performance found an association between the “executive control network” and TMT-B completion time scores (Seeley et al., 2007). In the study, it was reported that faster TMT-B performance (indexed by the completion time cost of TMT-B relative to the TMT-A) was associated with increased FC between bilateral intraparietal sulci and the remainder of the executive control network. More recently, a rs-FC study in a cohort of young, healthy adults extended these findings by highlighting the role of the FC between the left superior parietal lobule and right ventrolateral prefrontal cortex in mediating TMT switching performance (James et al., 2016). In this study, the TMT variant of the D-KEFS (Delis et al., 2001) was used to estimate the completion time increase for the letter-number switching condition (analogue of the TMT-B) relative to the number sequencing baseline condition (analogue of the TMT-A).

Others have used the TMT in conjunction with rs-FC to investigate the neural mechanisms of age-dependent executive decline (Damoiseaux et al., 2008). In line with several studies documenting an age-dependent decline in TMT-B performance (e.g. see Bowie and Harvey, 2006), significantly longer TMT-B completion times were observed in the older relative to the younger group. Separate correlational analyses were conducted between the raw TMT-B completion time scores and resting-state integrity of the anterior component of the default mode network (DMN) within each age group. A significant correlation was reported in the older, but not in the younger group. In particular, slower TMT-B performance was associated with lower anterior DMN integrity in the older group, suggesting that intrinsic connectivity strength within the anterior component of the DMN may contribute to the age-dependent decline in executive functions.

## 4 Neural correlates of the TMT: converging evidence and discrepant findings

Given the many processes recruited during performance of the TMT, it is likely that a network comprising functionally interconnected nodes, rather than a single brain region, better predicts performance. To identify the possible neural correlates of the TMT, we examine the brain regions most consistently reported in the VLSM and fMRI studies reviewed in the previous sections. We also discuss the primary factors that should be considered when evaluating discrepancies in the reported brain regions.

### 4.1 Synthesis of the brain regions linked to the TMT

**Fig. 1:**
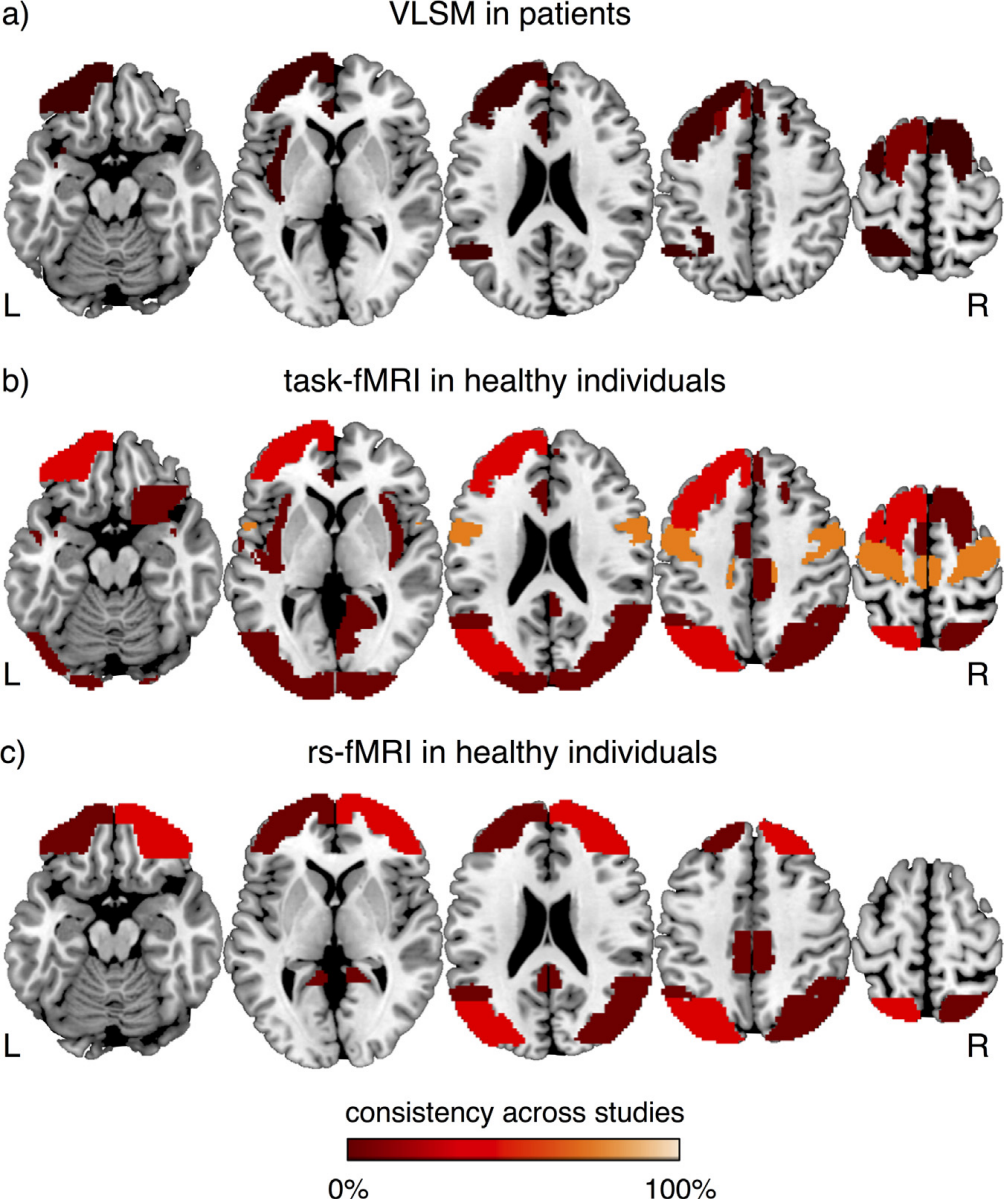
Brain regions identified by VLSM and fMRI studies of the TMT. The brain structures reported in VLSM (a), task-related fMRI (b) and resting-state fMRI (c) were mapped over the Harvard-Oxford brain atlas. The consistency of the neural effects was expressed in percentage, and calculated as the ratio between the number of studies that reported a brain region and the total number of reviewed studies.

The brain regions that, when lesioned, are associated with impaired TMT performance are variable across the VLSM studies (Table 1, Fig. 1a). Overall, the studies converge on demonstrating an association between left-lateralised lesion sites and poor performance on the TMT-B. Specifically, the lesion effects include regionally circumscribed sites within the left rostral anterior cingulate (Gläscher et al., 2012), left dorsomedial prefrontal cortex (Miskin et al., 2016) and left insular cortex (Varjacic et al., 2017), as well as regionally non-specific lesion sites within left prefrontal, insular, temporal and parietal cortex (Barbey et al., 2012). In addition, a right-lateralised effect was identified within the dorsolateral prefrontal cortex (Kopp et al., 2015).

Task-related fMRI studies of the TMT identified widespread neural effects (Fig. 1b). These included the left (Moll et al., 2002) and right (Jacobson et al., 2011) inferior/middle frontal gyrus, as well as superior and middle frontal areas (Zakzanis et al., 2005). Non-frontal activation effects were less consistently reported in relation to performance on the TMT-B, specifically in the bilateral superior parietal (Moll et al., 2002) and temporal areas (Zakzanis et al., 2005, Jacobson et al., 2011).

Resting-state fMRI studies of the TMT highlighted the importance of FC between the bilateral superior parietal cortex and prefrontal regions (Seeley et al., 2007, James et al., 2016) for adequate performance on the TMT-B (Fig. 1c). A rs-FC study suggested that intrinsic connectivity within the DMN, including superior and middle frontal gyri, posterior cingulate, and bilateral superior parietal cortex, mediated age-related executive decline, measured through the TMT (Damoiseaux et al., 2008).

Comparison of the reported neural effects across the TMT studies conducted in neuropsychological patients (Fig. 1a) and healthy individuals (Fig. 1b and c), revealed limited converging evidence (see also, MacPherson et al., 2015). No clear lateralization effects were identified, although areas in the left hemisphere were more frequently reported than areas in the right hemisphere. The regions that were found both in fMRI and VLSM studies were located in the prefrontal and superior parietal cortex. Future research is warranted to better qualify the regional specificity of these contributions.

### 4.2 Factors contributing to the variability across lesion-symptom mapping studies

A number of factors may explain the observed variability in the brain regions identified across lesion-symptom mapping studies.

#### 4.2.1 Sample aetiology

Aetiology is an important factor determining the underlying brain damage topography. Accordingly, the use of different aetiology subsets in the VLSM studies might have contributed to the regional variability of the identified lesion effects. The studies consecutively enrolling patients from a clinical setting tend to describe behavioural and neural effects in a more representative but heterogeneous group of patients compared to cohorts defined on the basis of the pre-specified criteria. For instance, Gläscher et al. (2012) employed an aetiology-diverse sample where the majority of patients were chronic stroke survivors, but they also included a limited number of patients with temporal lobectomy, focal surgical resection, encephalitis, and non-classifiable focal pathology subsets. Similarly, Miskin et al. (2016) used a relatively small and highly diverse sample of neurological conditions that included epilepsy, tumor and meningioma. Whereas collapsing across diverse aetiologies may be justifiable (Pa et al., 2010), systematic comparisons of behavioural and lesion effects across different aetiologies within studies are warranted. In contrast to the above studies employing heterogeneous brain-injured cohorts, some studies used a homogenous sample of patients. Notably, this choice may lead, in turn, to a limited sample size and limited spatial coverage as was the case, for instance, in a small sample study of right-hemispheric acute stroke patients with frontal lesion damage (Kopp et al., 2015).

#### 4.2.2 Dependent variables and their psychometric properties

With the exception of two studies that employed a variant of the standard TMT (i.e. the TMT variant of the D-KEFS in Barbey et al., 2012; the TMT variant of the OCS in Varjacic et al., 2017), the remaining VLSM studies administered the TMT using the standard protocol. Some VLSM studies employed raw TMT-B completion time (Miskin et al., 2016) and accuracy metrics (Kopp et al., 2015, Muir et al., 2015, Varjacic et al., 2017), whereas the other studies used ratio, proportional or difference scores (Gläscher et al., 2012, Muir et al., 2015, Varjacic et al., 2017, Barbey et al., 2012). These direct and derived TMT indices may differ in their psychometric properties. Importantly, the reliability of the available indices is thought to fall outside the desirable range (see Kopp et al., 2008, for an overview). Furthermore, different indices may tap onto different cognitive processes. Sánchez-Cubillo et al. (2009) indeed showed that performance on the TMT-A mainly reflects visuoperceptual abilities, performance on the TMT-B working memory and set-switching ability, whereas difference scores may be a relatively pure indicator of set-switching abilities (but see Kopp, 2011 for a discussion on the reliability of the difference scores).

Noteworthy, TMT is heavily reliant on intact number and letter sequencing processes. It may therefore be less suitable for detecting executive impairment in aetiologies typified by verbal and numerical impairment. If the standard TMT is administered, the score used for VLSM analyses necessitates correction for the presence of verbal/numerical disturbances. So far, only two studies that used brain-injured patients controlled for verbal impairment (Gläscher et al., 2012, Kopp et al., 2015). In particular, Gläscher et al. (2012) employed extensive correction of the (TMT-B *minus* TMT-A) completion time by parsing out the contributions of spatial abilities, visual and verbal memory.

### 4.3 Factors contributing to the variability across fMRI studies

The fMRI studies of the TMT differ to the extent they altered the TMT motor response modality. While the pioneering effort in TMT-fMRI adaptation stripped the TMT of the its visual and motor components (Moll et al., 2002), the follow-up studies either retained (Zakzanis et al., 2005) or attenuated (Jacobson et al., 2011) the motor properties of the task. This has made each of these fMRI adaptations different with respect to the psychological processes elicited by the task instructions framed either in terms of covert articulation (Moll et al., 2002), fast and accurate motor performance (Zakzanis et al., 2005) or fast and accurate orientation judgement (Jacobson et al., 2011). Below we summarise the aspects of the fMRI design of the TMT that makes the task depart from the standard administration protocol.

#### 4.3.1 Time constraints

The use of a time constraint might modulate TMT performance inside the MR scanner. Specifically, the optimisation of fMRI designs for the comparison of neural activation elicited by the TMT-A and TMT-B conditions requires embedding the task presentation within blocks that have a fixed duration. This requirement conflicts with the notion that the TMT is constructed to measure an added cognitive demand of the TMT-B via completion time increase in the TMT-B relative to the TMT-A condition. In the context of the fMRI studies, a completion time metric is rendered uninformative by the time constraint. The fMRI studies of the TMT thus tend to describe the behavioural effects either in terms of performance accuracy or by deriving a study-specific metric made available by a novel (i.e. fMRI-adapted) task context (e.g. “switch time”, Jacobson et al., 2011). In this respect, preliminary evidence suggests that the time constraint itself may modulate the behaviour during the TMT (Jacobson et al., 2011). Similar to the pc-TMT described above, the task involved linking up stimuli by indicating the orientation of the square attached to the target circle. The added response modality conferred the advantage of quantifying response time for each individual circle stimulus. Participants performed the task in timed and untimed task modalities, and behaviour was quantified in terms of an average response time. Significantly higher TMT-B response times were reported in the untimed relative to the timed condition. Notably, participants exhibited comparable at-ceiling accuracy levels across timed and untimed TMT-B conditions, suggesting that the time constraint made participants faster, but equally efficient. This finding suggests that the presence of the time constraint in the fMRI adaptations of the TMT may modulate behavioural performance, raising the possibility that the direction of this modulatory influence (performance improvement versus decrement) may depend on the study-specific task requirements of the fMRI-adapted TMT. This makes the result difficult to generalise to the performance recorded in the standard TMT task context, in which participants are typically instructed to perform the task as fast as they can.

#### 4.3.2 Participant’s adaptation/learning

The standardised TMT involves one-off and sequential task presentation of the TMT-A and TMT-B conditions. In this respect, there is some preliminary behavioural evidence suggesting that TMT-B performance is enhanced when the TMT-B occurs after the TMT-A, as opposed to when the presentation order of conditions is reversed (Takeda et al., 2011). The TMT-B performance “gain” due to prior TMT-A exposure (Takeda et al., 2011) should be emphasised in light of the repeated mode of presentation of the TMT conditions in the fMRI experiments. The above-mentioned fMRI studies included multiple repetitions of the TMT-A and TMT-B conditions, however, they did not statistically test for time-dependent performance changes.

## 5 Future directions

We propose that future efforts to characterize the neural basis of the TMT should be directed towards: 1) adopting a systematic approach for a reliable use of the TMT in both VLSM and fMRI studies; 2) defining a reliable and valid scoring system that enhances the sensitivity for unravelling the neural basis of the TMT; 3) examining TMT performance in relation to efficient information transfer (functional connectivity) between distant brain regions, both in the healthy and injured brain. We will elaborate on each of these research strands in the following sections.

### 5.1 Development of a TMT tool for VLSM and fMRI studies

We have suggested that the variability in the cognitive processes associated with different TMT versions may be a limitation for systematic and reproducible research on the neural correlates of the TMT. VLSM and FC studies rely on behavioural testing performed before or after the imaging session. The TMT used in these studies typically follows the standard administration protocol. In contrast, fMRI experiments require an extensive adaptation of the TMT. In brief, the TMT involves a strong motor component that is reflected in a highly coordinated and goal-directed drawing movement. The fMRI studies that either attempt to minimise (Jacobson et al., 2011) or eliminate (Moll et al., 2002) motor components inevitably alter the TMT response modality. A promising avenue to address these concerns is the development of an MR-compatible computerised TMT that would elicit psychological demands more closely matched to the standard TMT. A recent study presented evidence to suggest that tablet-based devices may be technically feasible for data acquisition inside the MR scanner (Tam et al., 2011). Another advantage of the tablet-based TMT is the ability to acquire fine-grained outcome measures, for instance the quantification of response time per connected step in the trail, in addition to the conventionally recorded total completion time. Future research should be aimed at assessing the clinical utility of the tablet-based TMT variants. This would provide the basis for a systematic study of the neural processes underlying the TMT by using functional imaging in healthy individuals as well as in patients with brain lesions. In the context of patient testing, the use of TMT variants instead of the standard TMT should also be carefully considered. For instance, a stroke-optimised TMT variant has been developed as part of the OCS battery (Demeyere et al., 2015, Varjacic et al., 2017). This TMT variant requires the participant to connect different shapes in alternating order. In this manner, the derived TMT measures are not confounded by the possible presence of verbal and numerical impairments occurring after brain injury.

### 5.2 Optimisation of the TMT-derived scores for neuroimaging studies

Previous studies showed the validity of the TMT for probing set-switching ability (e.g. Arbuthnott and Frank, 2000; but see Sánchez-Cubillo et al., 2009). Several aspects should however be considered when evaluating the pattern of neural effects underlying the performance on the TMT. For instance, the sensitivity of the TMT to detect frontal lobe damage may depend on the outcome variable (completion time versus accuracy versus error type) as well as the correction applied to the TMT-B completion time scores. In particular, the TMT-B scores may be sensitive to frontal brain damage (Delis et al., 2001, Ettlin et al., 2000), assuming that the chosen TMT index is a reliable indicator of set-switching ability. This raises the question of which outcome variable and correction type performs better at isolating the set-switching component of the TMT-B. Based on these considerations, a recommendation for future research involves the assessment of the reliability and construct validity of direct and derived TMT indices (incl. completion times, accuracy measures and error types) in both healthy volunteers and brain-injured patients.

### 5.3 Using novel neuroimaging techniques to study the neural correlates of the TMT

The heterogeneity of the reported neural effects may not only be attributed to the different approaches used for measuring performance, but potentially also to a limited adequacy of regional neural measurements to capture executive demands of the TMT. Based on the reviewed studies (see Fig. 1), we posit that the neural underpinnings of the TMT may be better investigated at the level of large-scale brain networks. An avenue for future research might specifically rely on recent developments in the field that involve the use of resting-state fMRI in patient cohorts (Aerts et al., 2016, Gillebert and Mantini, 2013). More specifically, rs-FC measures can be used for the characterisation of the altered network organisation following focal brain injury and for establishing the link with executive function deficits assessed through the TMT. One approach for the quantification of network reorganisation is grounded on the idea that the brain tends to exhibit a modular structure of its constituent subnetworks (e.g. Gratton et al., 2012). The use of modularity metrics (Newman and Girvan, 2004, Gratton et al., 2012) may shed light on the way brain injury alters the functional architecture of the brain. Within this framework, regions with a high number of between-module connections can be conceived as facilitating communication between individual sub-networks (referred to as “connectors”), in contrast to regions characterised by a high degree of within-module connections (referred to as “hubs”). Lesion masks may be incorporated in the analysis by quantifying within-module and between-module connections for each damaged node (i.e. lesion), such that damage scores can be derived for connectors and hubs, respectively. Such an approach may be useful to test whether reduced TMT executive performance in brain-injured patients is more related to damage scores for connectors than for hubs. This investigation may corroborate the hypothesis that the deficits observed in the TMT can be better explained in terms of inefficient exchange of information between distant brain regions, rather than in terms of local damage at specific cortical locations.

Another recent development in the field of FC involves the direct comparison between individual patients and a normative sample of healthy individuals. For instance, the voxels belonging to the structural lesion in a brain-injured patient can be used as seeds in the FC analyses. The statistical comparison between the FC map in the patient against the corresponding FC maps from a normative sample (Siegel et al., 2014, Boes et al., 2015) permits the delineation of brain regions with altered FC. These brain regions may be found at a distance from the structural lesion, which could be explained in terms of diaschisis and other compensatory processes (Carrera and Tononi, 2014). The utility of the lesion-derived FC approach may be further enhanced by its joint application with VLSM (Sutterer et al., 2016). This analysis extends VLSM inferences regarding the role of isolated structural lesions in predicting behavioural deficits, by identifying patterns of aberrant FC stemming from these lesions in association with a behavioural deficit of interest (for instance, see Sutterer et al., 2016 for a study on decision making). Future studies combining VLSM and lesion-derived FC approaches are warranted to map TMT scores onto network-level disturbances. In this regard, previous VLSM research on the TMT has laid the groundwork for this type of analysis by identifying patterns of discrete structural damage in association with the TMT-indexed deficits. This can support the a priori selection of seed regions in studies using a combination of the lesion-derived FC maps and VLSM.

## 6 Conclusions

We have examined neuroimaging studies conducted in healthy individuals and in brain-injured patients, to identify the neural correlates of the TMT. These studies generally indicate that large-scale brain networks including prefrontal and parietal structures mediate TMT performance. Both VLSM and functional neuroimaging studies of the TMT however reported a heterogeneous pattern of structural damage and neural activation effects. In this review, we suggest that this may be explained, at least in part, by the approach used for behavioural testing, the participants’ selection criteria, and the analyses used for linking brain imaging and behavioural data. Relying on advanced technical solutions may be important for an optimal use of the TMT in neuroimaging studies and in particular for bridging the gap between VLSM studies in patients and fMRI studies in healthy individuals. We suggest that tablet-based versions of the TMT could be employed not only in clinical settings, but also in the MR scanner, allowing for more refined and reliable measures to be used for brain-behaviour correlation analyses. Importantly, TMT performance measures in brain injured-patients can be linked to FC indices derived from resting state fMRI data, to test whether behavioural impairments following brain damage are mediated by altered connectivity in brain networks. The identification of the brain networks involved in TMT performance may also help clarifying whether the TMT can be used as a primary measure of set-switching ability.
